# Developing a Method to Quantify Swelling Force Generated by the Osmotic Pump Tablet Push Layer †

**DOI:** 10.3390/pharmaceutics18091174

**Published:** 2026-09-17

**Authors:** True L. Rogers, Stephanie Robart, Thomas Watson, Rhea Wang, Harold Bernthal, Vahid Ahmadi

**Affiliations:** Roquette Health & Pharma Solutions (HPS), Midland, MI 48642, USA; stephanie.robart@roquette.com (S.R.); thomas.watson@roquette.com (T.W.); rhea.wang@roquette.com (R.W.); vahid.ahmadi@roquette.com (V.A.)

**Keywords:** push–pull osmotic pump (PPOP) tablet, push layer, polyethylene oxide (PEO), osmotic pressure, dimensional expansion, axial swelling, swelling force, controlled release

## Abstract

**Background/Objectives**: A push–pull osmotic pump (PPOP) tablet is a controlled-release delivery system consisting of a bilayer tablet (push layer/pull (drug) layer), coated within a selectively permeable barrier membrane, and containing a laser-drilled orifice in the pull layer side of the barrier. Aqueous media permeates across the barrier membrane and into the bilayer tablet core. The push layer swells axially, creating force and acting like a piston to drive the active pharmaceutical ingredient (API) dosage through the laser-drilled orifice. The primary purpose of this study was to develop a method to quantitate swelling force generated by the PPOP push layer. **Methods**: A key challenge was devising measurement methodology that most closely represented continuous PPOP push-layer swelling force exerted in the axial dimension over time, so the method setup was adjusted until attaining what closely approximated PPOP push-layer swelling-force dynamics within the delivery system. After some adjustments, swelling forces were measured from PPOP push layers containing various polyethylene oxide (PEO) molecular weight (MW) grades. **Results**: The most representative configuration was a cylindrical holder fully submerged in water with the push-layer compact situated at the bottom of the holder. This setup constrained the push layer to swell in the axial dimension. The optimal setup also allowed the texture analyzer to continuously measure push-layer swelling force over 24 h, representing typical duration of functionality. Quantitative analysis of the swelling force vs. time profiles demonstrated comparable force generation over 24 h from the 4, 5, and 7,000,000 MW grades of PEO. Swelling force decreased in near-linear fashion, from push layers formulated with PEO 4,000,000 down to PEO 100,000. **Conclusions**: The optimal setup enabled discriminatory quantification of push-layer swelling force for PEO MW grades spanning from 100,000 up to 4,000,000 Daltons. Furthermore, the method demonstrated why the 4, 5, and 7,000,000 MW grades of PEO are recommended for use in the PPOP push layer, given that these three highest MW grades delivered comparable swelling force over 24 h. Finally, the method provides new quantitative insight into the push-layer swelling force that is necessary, over the duration of the dosing interval, to deliver the active dosage from the PPOP tablet.

## 1. Introduction

**Figure 1:**
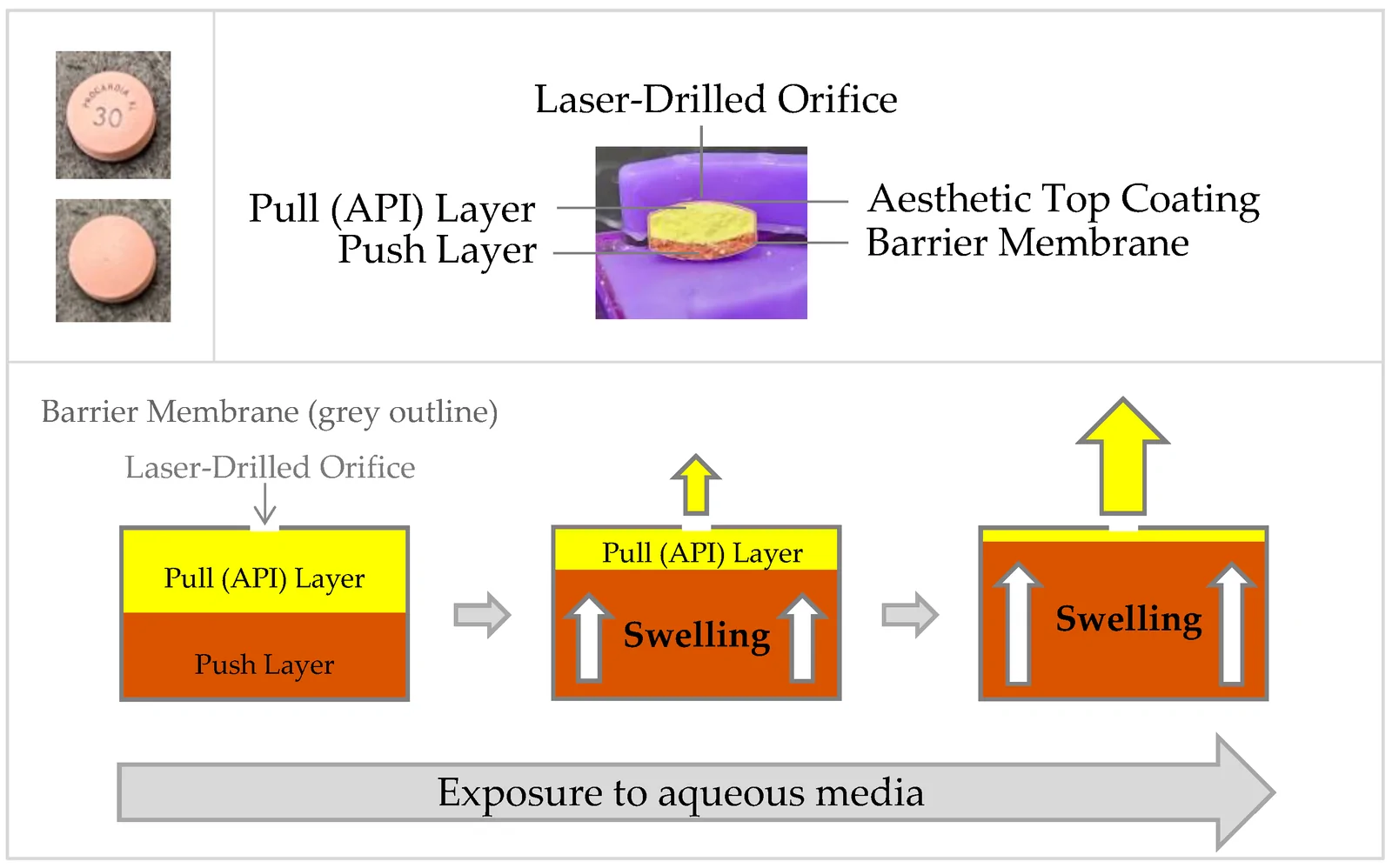
Illustration of PPOP tablet and how it functions. PPOP tablets in the top half of the figure are Procardia XL 30 mg [4].

Push–pull osmotic pump (PPOP) tablets are controlled-release oral solid dosage forms (OSDFs) consisting of a bilayer tablet core coated within a selectively permeable barrier membrane [1,2,3]. PPOP tablets represent an established and robust controlled-release delivery system with several marketed examples [1], some of which are listed in Table 1. Procardia XL [4], shown in Figure 1, is a once-daily PPOP tablet that releases nifedipine over 24 h via zero-order kinetics for much of the dosage interval. Nifedipine is a calcium channel blocker used to treat cardiovascular conditions, such as hypertension. Hormonal fluxes, which coincide with the circadian rhythm, cause blood pressure to fluctuate throughout the day. Blood pressure surges are known to occur in the early morning hours before waking [5], so it is crucial that nifedipine is still being released during those final hours of the dosage interval prior to waking and administering the next dose.

In simple terms, as illustrated in Figure 1, the pull layer contains the active pharmaceutical ingredient (API) and a relatively low molecular weight (MW) polymeric excipient as carrier for the API dosage. The push layer contains a high-MW polymeric excipient, osmogen, and pigment. The osmogen creates osmotic pressure, which facilitates aqueous media permeation into the bilayer core. The pigment allows differentiation between the push and pull layers, along with correct orientation of the coated bilayer tablet for laser-drilling the delivery orifice on the pull-layer side of the barrier membrane. Once exposed to aqueous media, the media permeates across the barrier membrane into the bilayer core through osmosis [6]. The aqueous media causes the pull layer to become more mobile and fluid-like and the push layer to swell [7]. The push layer is constrained to swell in the axial dimension due to the confined space within the barrier membrane coating, which is sufficiently durable to avoid rupture from internal swelling [8]. Axial swelling from the push layer essentially acts as a piston to extrude the mobile pull layer out of the laser-drilled orifice in controlled fashion. As will be demonstrated, the push layer must provide sustained swelling force that is sufficient to drive API release from once-daily PPOP tablets, like Procardia XL [4], over the entirety of the dosage interval.

Osmotic pressure is a solute-solution colligative property and is the pressure required to prevent water molecules from traversing from low to high solute concentration. In contrast, swelling force derives from pressure generated by dimensional expansion. Osmotic pressure and swelling-force mechanisms operate concomitantly following introduction of a PPOP tablet into an aqueous environment. Aqueous media enters the PPOP tablet via osmosis across the semipermeable membrane, and the push layer expands to generate force as the high-MW polymer swells. A key objective of our study was to decouple the influence of dimensional swelling force from osmotic pressure and to measure push-layer swelling force over a timeframe representative of a typical PPOP tablet dosage interval.

We searched the literature for fundamental approaches to quantifying mechanistic functionality of push-layer swelling force. Two primary approaches were identified: osmotic pressure measurement [9,10,11] and dimensional (swelling) visualization/quantitation [12,13,14,15,16,17].

Many of the studies found in the literature focused on push-layer dimension dynamics and influence of polymer molecular weight on extent of dimensional change; however, we found none directly quantifying push-layer swelling force over a representative duration of controlled release, such as 24 h. In the most closely relevant study, Chen et al. visualized dimensional swelling over time and then measured mechanical strength of the push-layer gel at comparable swollen dimensions. Using this approach, push-layer mechanical strength directly correlated with PEO molecular weight, such that higher MW generated greater gel strength [13].

The swelling polymer in the PPOP push layer is typically high-MW polyethylene oxide. The 4, 5, and 7,000,000 Dalton MW grades of PEO have been reported to deliver comparable controlled-release performance from PPOP tablets [13,18,19]. Thus, push-layer compacts were formulated first with PEO 5,000,000, NaCl as osmogen, and iron oxide as pigment, and these push-layer compacts were used to develop and hone the methodology parameters that would most closely represent axial swelling of the push layer inside the PPOP tablet over time. Once methodology was established, push layers were formulated containing the 4 and 7,000,000 MW grades of PEO. Although PEO MW grades lower than 4,000,000 are not normally used in the PPOP push layer, MW grades from 2,000,000 down to 100,000 were also formulated and characterized to determine the discriminatory capability of the new methodology. This paper expands on initial research presented at the AAPS PharmSci360 conference in 2025 [20] and documents method development, as well as quantitation, of PPOP push-layer swelling force over 24 h duration and across a range of PEO MW grades.

## 2. Materials and Methods

Dissolution testing of the commercial 30 mg strength PPOP tablets was conducted in replicates of 7 (*n* = 7) in 900 mL of degassed 0.05% polysorbate 80 aqueous media, equilibrated to 37 °C. The PPOP tablet was secured in a hanging basket, as reported in [21]. Samples of 3 mL volume were collected at 0.5, 1, 2, 4, 6, 8, 10, 12, 15, 16, 20, and 24 h. Each collected sample was diluted in half with methanol and mixed to maintain nifedipine in solution, and then filtered through a 0.45 µm membrane. To quantify dissolved nifedipine, 20 µL of each filtered solution sample was injected via autosampler into an Agilent 1260 Infinity high-performance liquid chromatography (HPLC) instrument (Waldbronn, Germany) equipped with an ultraviolet (UV) detector (225 nm wavelength). The HPLC column (Proshell 120 EC C18 4 µm, 4.5 × 150 mm) was equilibrated to 40 °C, and a gradient method was used to separate nifedipine. From 0–15 min, the mobile phase consisted of 80% deionized water/methanol (90:10) mixture and 20% acetonitrile. From 15 to 20 min, the mobile phase consisted of 80% acetonitrile and 20% deionized water/methanol (90:10) mixture.

PPOP push-layer compacts consisted of 79.5% PEO, 19.5% micronized NaCl, and 1% red iron oxide. Information on these ingredients are listed in Table 2. Powders were mixed for 10 min at 10 g blend size using a Turbula mixer (Willy A. Bachofen AG (WAB Group), Muttenz, Switzerland). Blended powder was hand-fed into the die of 10.3 mm, round, flat-face beveled-edge (FFBE) tooling (Natoli Engineering Company, Inc., St. Charles, MO, USA), which was fitted in the 16-station turret of a Manesty Beta Rotary Press (Syntegon Technology, Baden-Württemberg, Germany). The push-layer compact weight target was 400 mg. The compression force target was 17.8 kN. The turret was operated at 12–14 rpm to compact the push layers. Push-layer compacts were allowed to equilibrate at least overnight prior to characterization.

**Figure 2:**
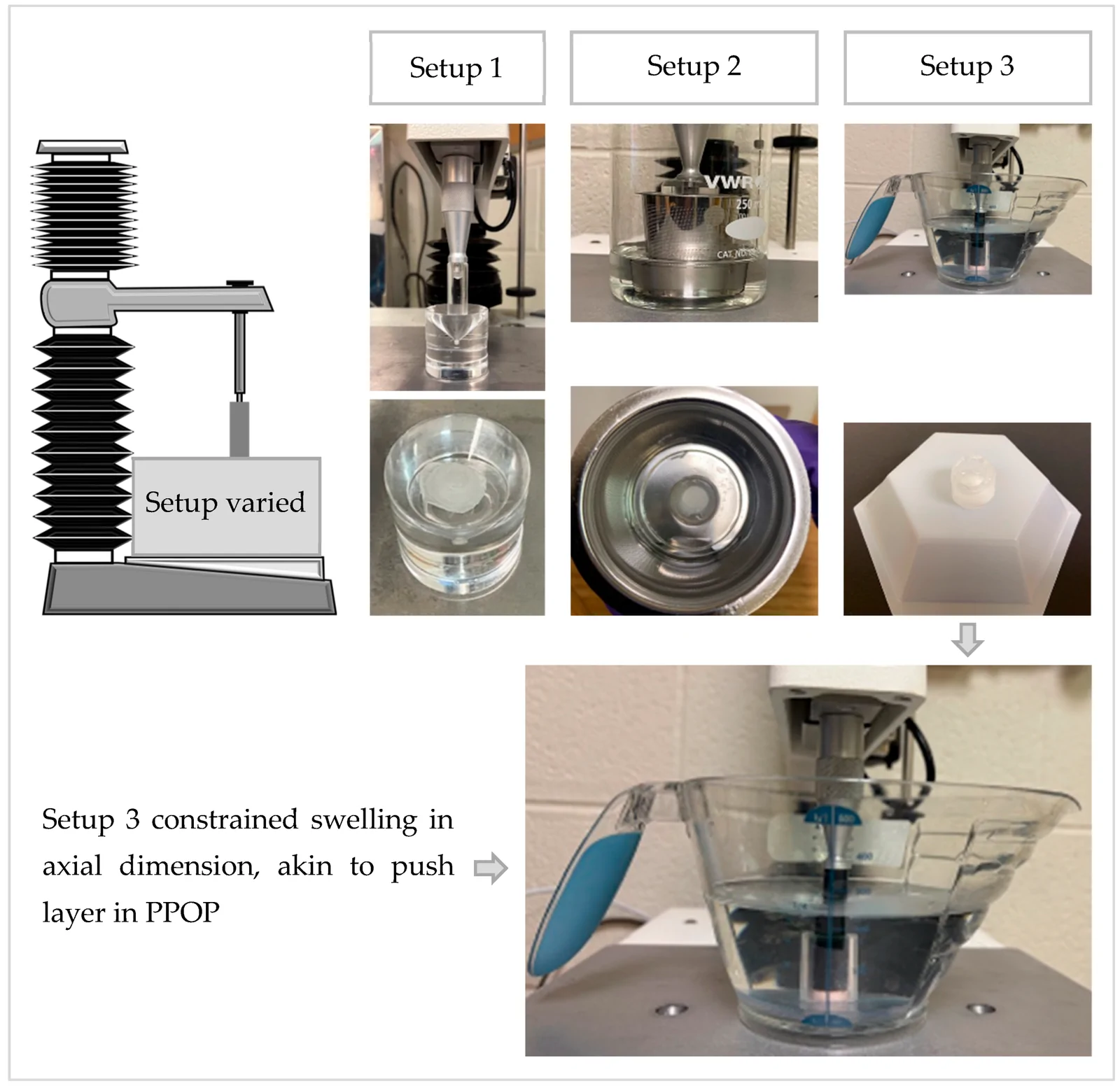
Texture analysis illustration by Knarr [22], but showing that setup and the push-layer compact’s configuration were adjusted to isolate measurement of swelling force in the axial dimension.

Swelling force was measured using a TA-XTplusC Texture Analyzer (Stable Micro Systems Ltd., Godalming, Surrey, UK), fitted with a 5 kg load cell and a flat-faced cylindrical probe of 10 mm diameter. Configurations for tablet position and water volume were varied, as shown in Figure 2. The probe was lowered to the point at which it was almost touching the push-layer compact. While lowering the probe down to the push-layer compact, care was taken to avoid both premature contact with the compact and contact with the setup holding the push layer in place. The measurement was initiated using a trigger force of 2 g. Once that force was attained, water was added to initiate push-layer swelling. The probe was constantly held at a static position, so the measured force was that generated as the push layer swelled in the axial dimension, pressing up against the static probe. Swelling force was measured at a rate of one data point per second over a 24 h duration, representative of the timeframe over which the PPOP tablet typically would provide controlled-release performance.

**Figure 3:**
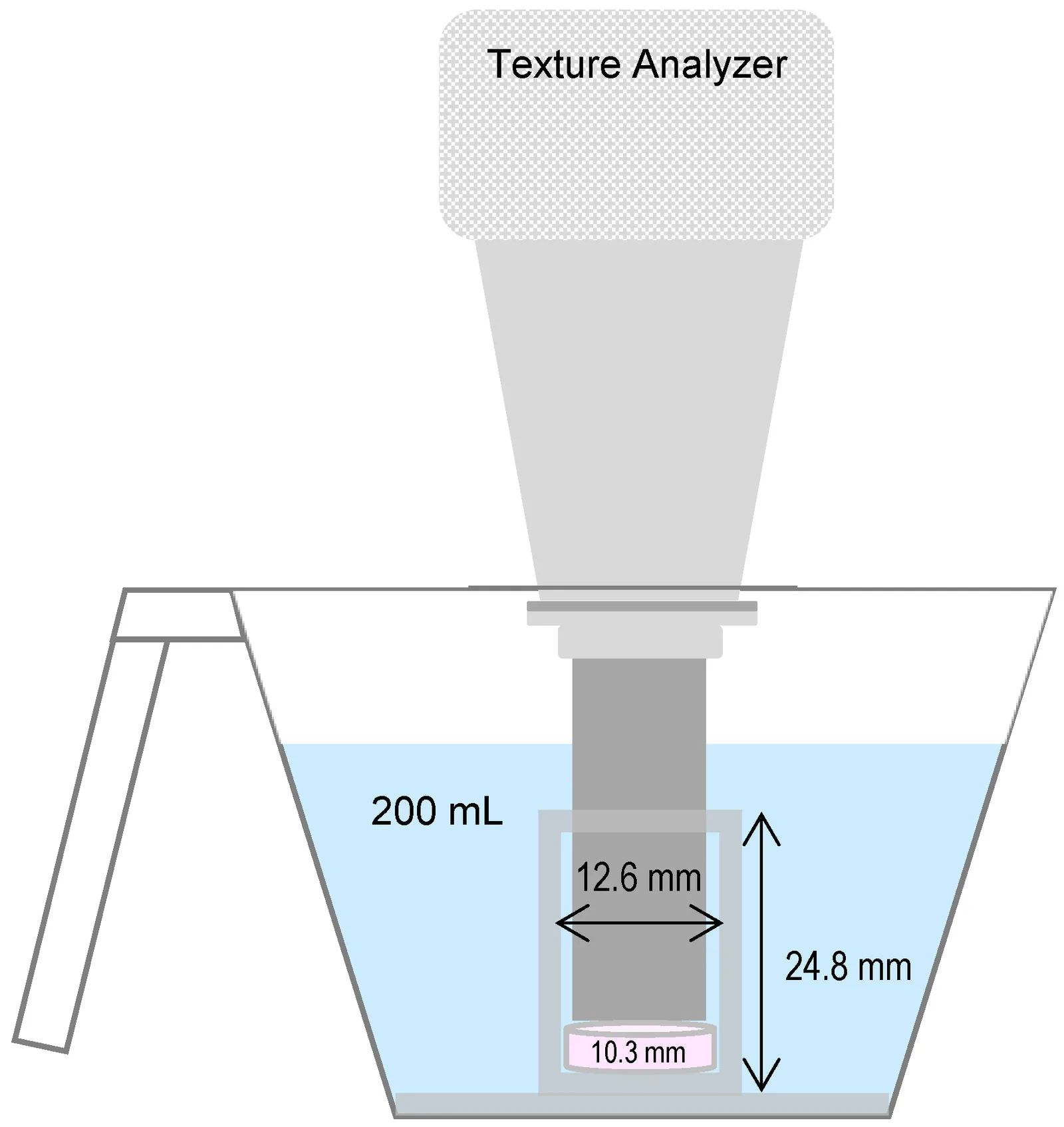
Detailed illustration of Setup 3.

The first setup with the push-layer compact situated in the conical cup had capacity for ~5 mL of water, and the entire 5 mL volume was consumed prior to completion of the 24 h swelling test. That is, there was insufficient volume of water to characterize the push layer using Setup 1. The second setup encompassed a perforated stainless-steel K-cup mesh, with the lower half of the K-cup submerged in 50 mL of water, all contained within a 250 mL beaker. Setup 2 did not adequately represent the confined space within the PPOP tablet because swelling eventually took the path of less resistance, transitioning from swelling in the axial dimension to swelling in the radial dimension. Consequently, the swelling-force measurement became unreliable due to dimensional swelling transition. The third setup consisted of a fabricated cylindrical design, made from plastic, and fitted snuggly into a 500 mL plastic measuring cup. The inner and outer diameters of the cylinder were 12.6 and 15.9 mm, respectively. The height of the cylinder was 24.8 mm. A volume of 200 mL of water was used with Setup 3 to ensure that the level of water would be of sufficient height to fully submerge the cylindrical holder and traverse down the narrow gap between the texture analyzer probe and the inside of the cylinder wall to access the push-layer compact’s perimeter continuously over 24 h. See Figure 3 for a more detailed schematic of Setup 3. Setup 3 provided the confined space to force swelling in the axial dimension and prevent swelling in the radial dimension, akin to how the push layer swells inside the PPOP tablet, so this setup was chosen for further investigation.

Computational and statistical analyses were conducted using Microsoft Excel for Microsoft 365 MSO (Version 2502, Microsoft Corporation, Redmond, WA, USA) and JMP 18.1.0 (SAS Institute Incorporated, Cary, NC, USA). Microsoft Excel was used to generate the swelling-force profiles, identify maximum swelling forces, and calculate areas under the swelling-force curves from 0 to 15 h and 0 to 24 h, but the computational limits of Excel were challenged by such large datasets. Hence, further statistical analyses were conducted in JMP via analysis of variance (ANOVA) and comparing means using Tukey’s honestly significant difference (HSD) test at α = 0.05 to mitigate family-wise error rate. Differences between datasets were considered statistically significant at *p* < 0.05. The datasets statistically analyzed in JMP included swelling forces at individual time points, namely 4, 8, 12, 15, 16, 18, 20, and 24 h; swelling-force maxima; areas under the swelling-force profiles from 0 to 15 h and 0 to 24 h; and differences between the areas under the swelling-force profiles from 0 to 15 h and 0 to 24 h.

## 3. Results

### 3.1. Drug Release

**Figure 4:**
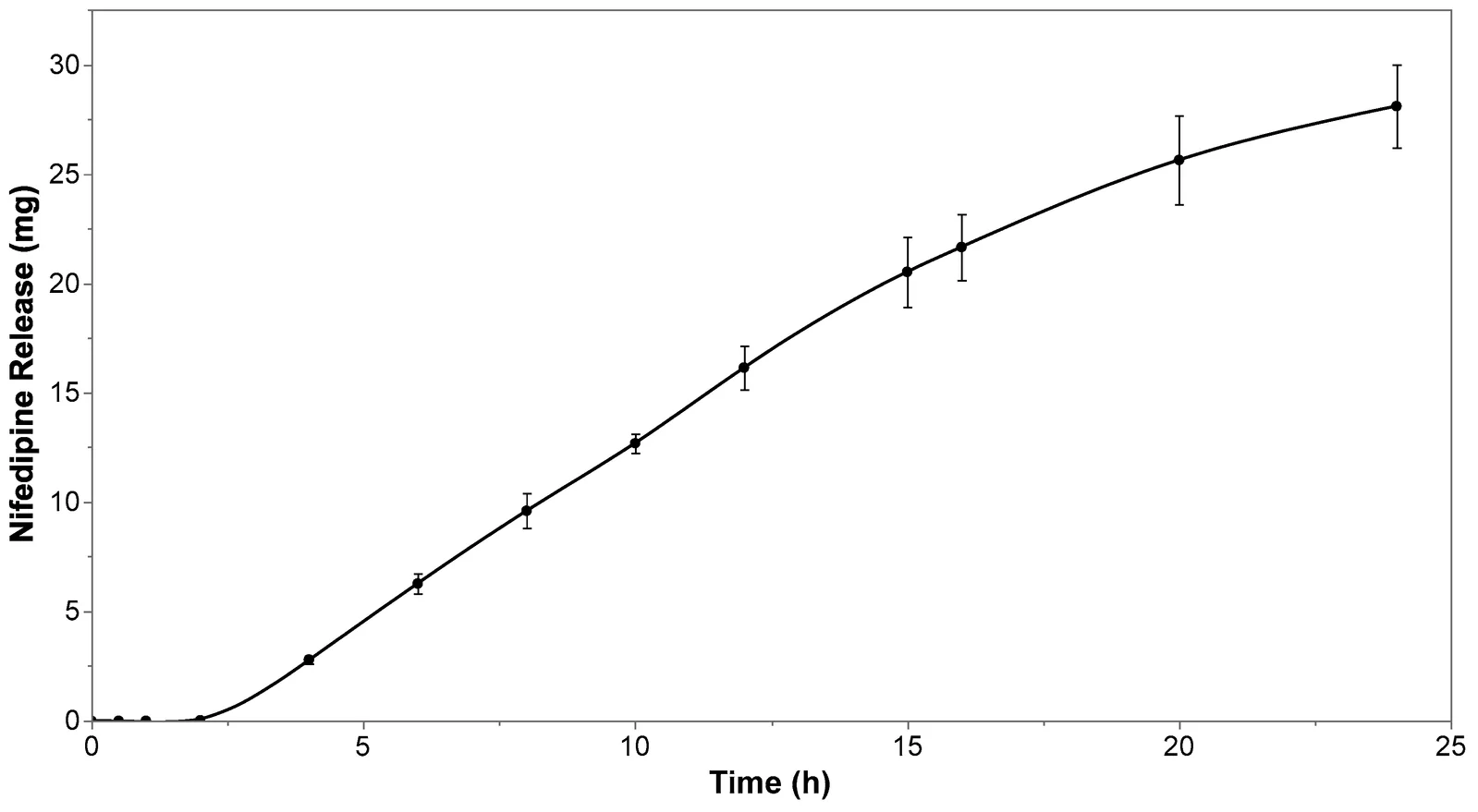
Controlled release of nifedipine from the 30 mg strength PPOP tablet over 24 h.

After the initial 2.5 h lag, controlled release of nifedipine from the 30 mg strength PPOP tablet occurred over the remainder of the 24 h test (Figure 4), following zero-order release kinetics from 2.5 to 15 h. Transition from zero-order to first-order release kinetics occurred between 15 and 16 h and continued in first-order fashion for the remaining 8 h. That is, by 15 h, 21 mg of the 30 mg nifedipine dosage had been released from the PPOP tablet. By 24 h, 28 of the 30 mg had been released.

The remainder of the study was dedicated to developing methodology that could measure the swelling force exerted by the PPOP push layer, known to drive the above-described controlled-release performance over the dosage interval.

### 3.2. Configuration Setup

Setup 1 (Figure 2) did not hold sufficient volume of water to allow for continuous hydration and swelling of the push layer over the duration of the test. Within a few hours of test initiation, push-layer swelling was visibly evident, and force was registering against the texture analyzer probe, but the tablet had not yet hydrated to its fully swollen state and was still absorbing water. At some point while the test ran overnight, the entire volume of water was absorbed by the push layer, and it had stopped swelling. The push layer had softened to the consistency of candlewax and had assumed the shape of the conical cup. A larger volume of water was needed, so we proceeded to Setup 2.

**Figure 5:**
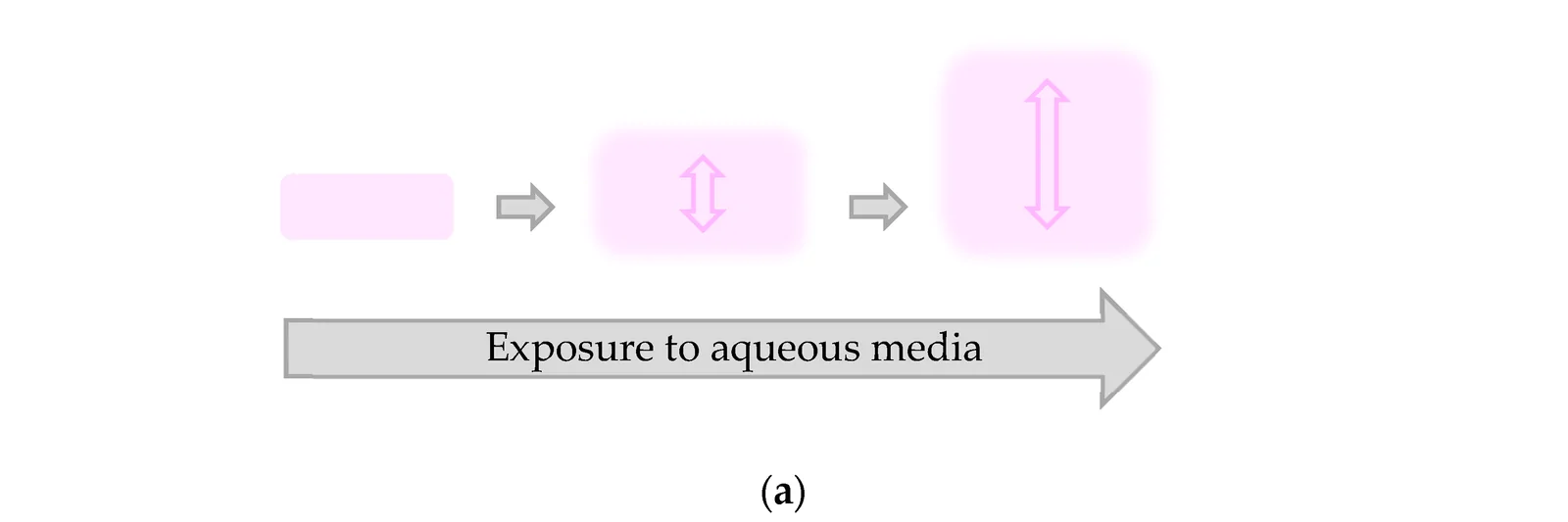

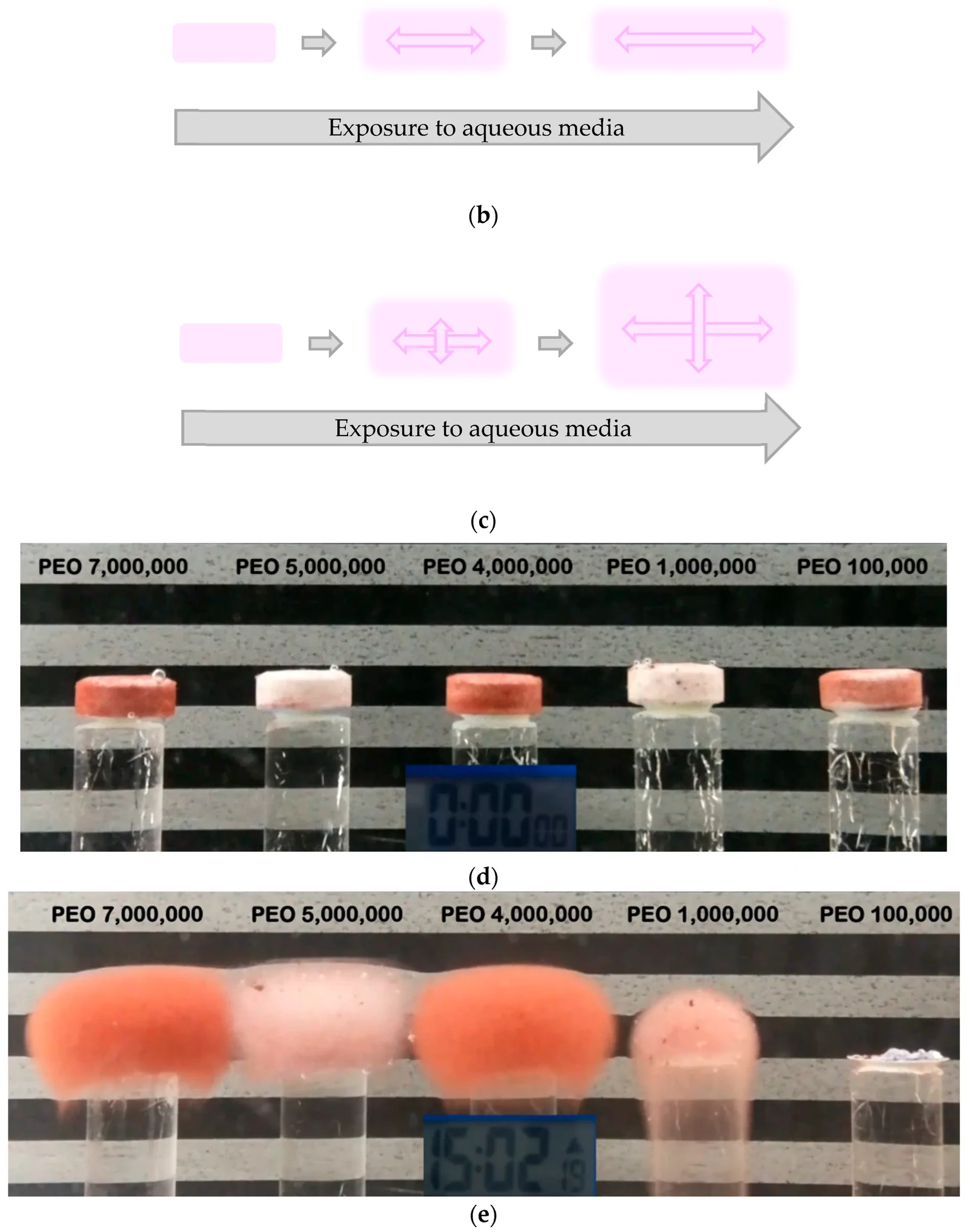
Visual illustration of axial (vertical) and radial (lateral) swelling of PPOP push layers. Graphical illustrations of swelling in the axial (**a**), radial (**b**), and both axial and radial dimensions (**c**). (**d**) Images of push layers containing different PEO MW grades at initial timepoint just after introduction to water. Timestamp in (**e**) indicates that this image was captured just over 15 h after introduction of the push layers to water. Push layers in (**d**,**e**) were not barrier membrane-coated, so swelling occurred in both axial and radial dimensions.

There was sufficient volume of water with Setup 2 to allow for continuous push-layer swelling over the 24 h testing duration, but there was too much space radially between the push layer and the K-cup wall. For the first few hours, force registered against the texture analyzer probe as the push layer absorbed water and swelled, but as more water was absorbed over the duration of the test, the push layer transitioned from swelling in the axial dimension towards taking the path of less resistance by swelling in the radial dimension (see Figure 2, Setup 2). With no radial boundaries, Figure 5 shows that the push layers will swell in both axial and radial dimensions. In PPOP tablets, swelling in the radial dimension is hindered by the thick, semipermeable barrier membrane and rather encouraged to occupy axial space made available as the pull layer exits through the laser-drilled orifice.

Ideally, Setup 3 would most closely approximate push-layer swelling within the PPOP tablet by forcing swelling to occur in the axial dimension. As pictured in Figure 2 and illustrated in Figure 3, the inner diameter of the plastic cylinder for Setup 3 was 2.3 mm greater than the diameter of the push-layer compact. This allowed sufficient clearance for the push layer to fit snugly and level at the bottom of the cylinder. The inner diameter of the plastic cylinder was 2.6 mm greater than the diameter of the texture analyzer probe, which allowed for continuous access of water to the push layer via the gap between the probe and the cylinder wall. Furthermore, it was necessary to avoid contact between the texture analyzer probe and the cylinder wall in order to circumvent measurement interference.

With this setup, the data measured would originate from push layer swelling in the axial dimension and exerting force against the statically positioned texture analyzer probe. The swollen push-layer tablet shown in Figure 2, Setup 3, is the push layer after completion of the 24 h swelling-force measurement. Upon close visual inspection, it is evident from its swollen geometry post-test that the push layer was confined to swell solely in the axial dimension. There was a slight extent of swelling up into the gap between the texture-analyzer probe and the inner wall of the cylindrical-tablet holder. We acknowledge with Setup 3 that a minor extent of hydrogel erosion could have occurred from that swollen push layer expanding into the gap, but we assumed erosion was minimal based on visual observation of the integrity of the push layer’s shape post-test (see Figure 2). Even the portion of the swollen push layer that expanded into the gap maintained its geometric integrity following the 24 h measurement, at least for those push layers containing the high-MW PEO grades.

### 3.3. Swelling Force

**Figure 6:**
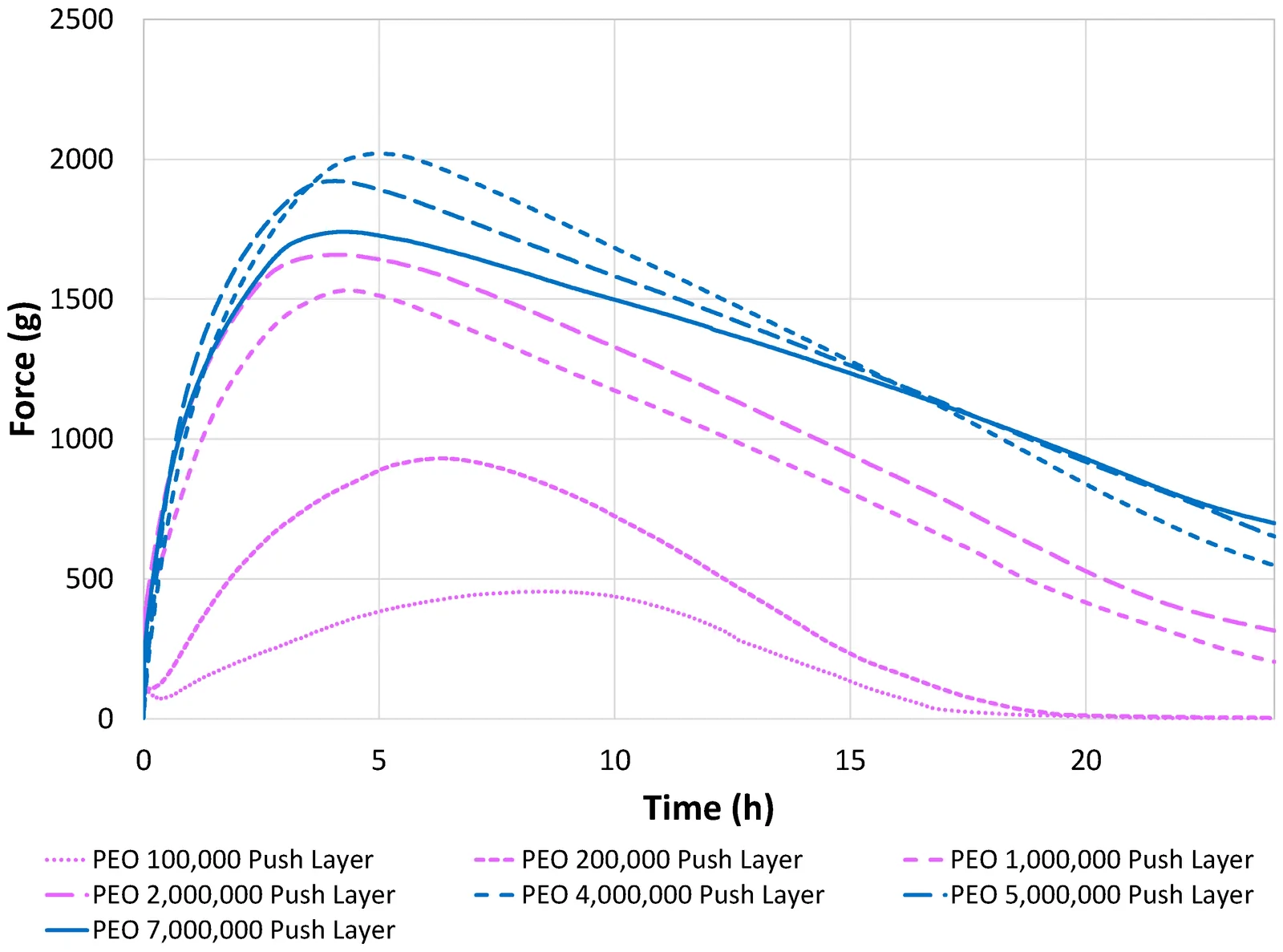
Average (*n* = 7) swelling force vs. time profiles from push layers formulated using different PEO MW grades.

The remainder of this paper is dedicated to measurements and analyses of push layers, with Setup 3 using different MW grades of PEO as swelling polymer. First, swelling force was measured vs. time with push layer formulated using PEO 5,000,000. Tests were repeated on seven separate push-layer compacts (*n* = 7). Next, push-layer compacts were formulated with PEO 4,000,000 and then with PEO 7,000,000; and both sets were measured in replicates of *n* = 7. The swelling force vs. time profiles from the three highest PEO MW grades are shown in blue in Figure 6. Swelling force vs. time was comparable from push layers containing these three highest MW grades, supporting the recommended use of these in the push layer.

The swelling force vs. time profiles from the lower PEO MW grades are shown in pink in Figure 6. The swelling-force profiles decreased in magnitude from PEO 4,000,000 down to PEO 100,000. Except for the two lowest MW grades, the swelling-force profiles peaked within 4–5 h. Push layers containing PEO 1,000,000 and higher delivered some extent of swelling force over the entire 24 h testing duration, with swelling force gradually waning in linear fashion, following peak-force attainment, over the remainder of the test. In contrast, push layers containing PEO 100,000 and 200,000 peaked within 6 to 8 h, followed by rapid decline in swelling force. No swelling force was detected after 16–18 h from push layers containing these two lowest PEO MW grades.

Considering the controlled-release profile in Figure 4, push-layer swelling force is needed to drive nifedipine release from the PPOP tablet over the entirety of the dosage interval. Recall that the transition from zero-order to first-order release kinetics occurred between 15 and 16 h, with the last 9 mg of the 30 mg nifedipine dosage released over those final 9 h of the dissolution test. Hence, those last 9 h of the swelling-force profiles in Figure 6, particularly for the three highest PEO MW grades (blue profiles), coincide with release of the final 9 mg of the dosage by the end of the dosing interval. Although this is the first time, to our knowledge, that swelling force from the PPOP push layer has been reported, the results aligned with what was expected: The overall push-layer swelling profile increased in magnitude with increasing PEO MW grade, and the three highest PEO MW grades delivered comparable swelling-force profiles over the 24 h duration, corresponding to their recommended use.

**Figure 7:**
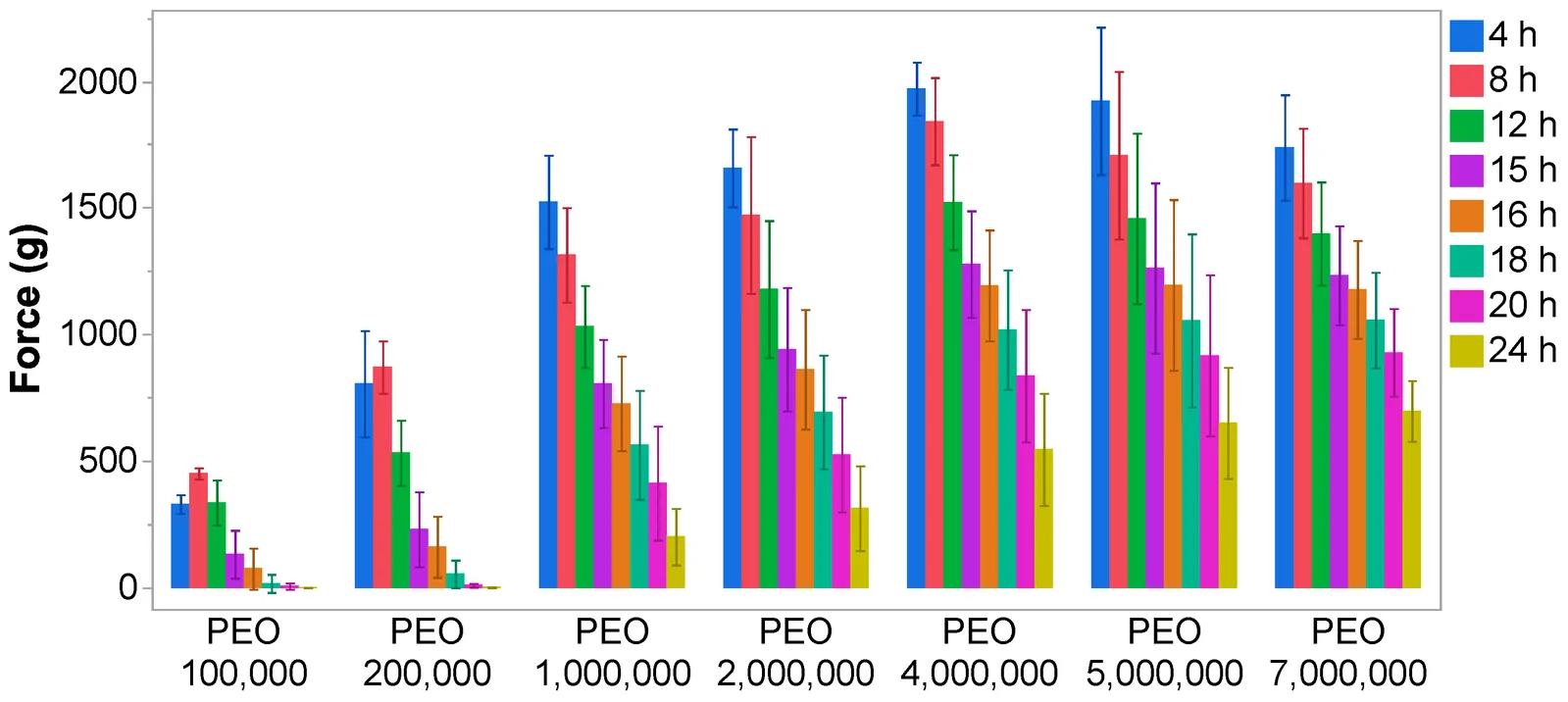
Swelling forces at specified time points from push layers formulated with different PEO MW grades. Each bar represents the average swelling force at the designated time point ± standard deviation (*n* = 7).

Because texture analysis measurements occurred at a rate of 1 datapoint per second over the 24 h test duration, we encountered computational memory constraints that prevented some statistical analyses in Microsoft Excel. Hence, swelling forces from a subset of time points are shown in Figure 7. Figure 7 exemplifies the increase in swelling force from push layers containing increasing MW grades from PEO 100,000 up to PEO 4,000,000, followed by similarity in swelling forces attained from push layers containing the 4, 5, and 7,000,000 MW grades.

**Figure 8:**
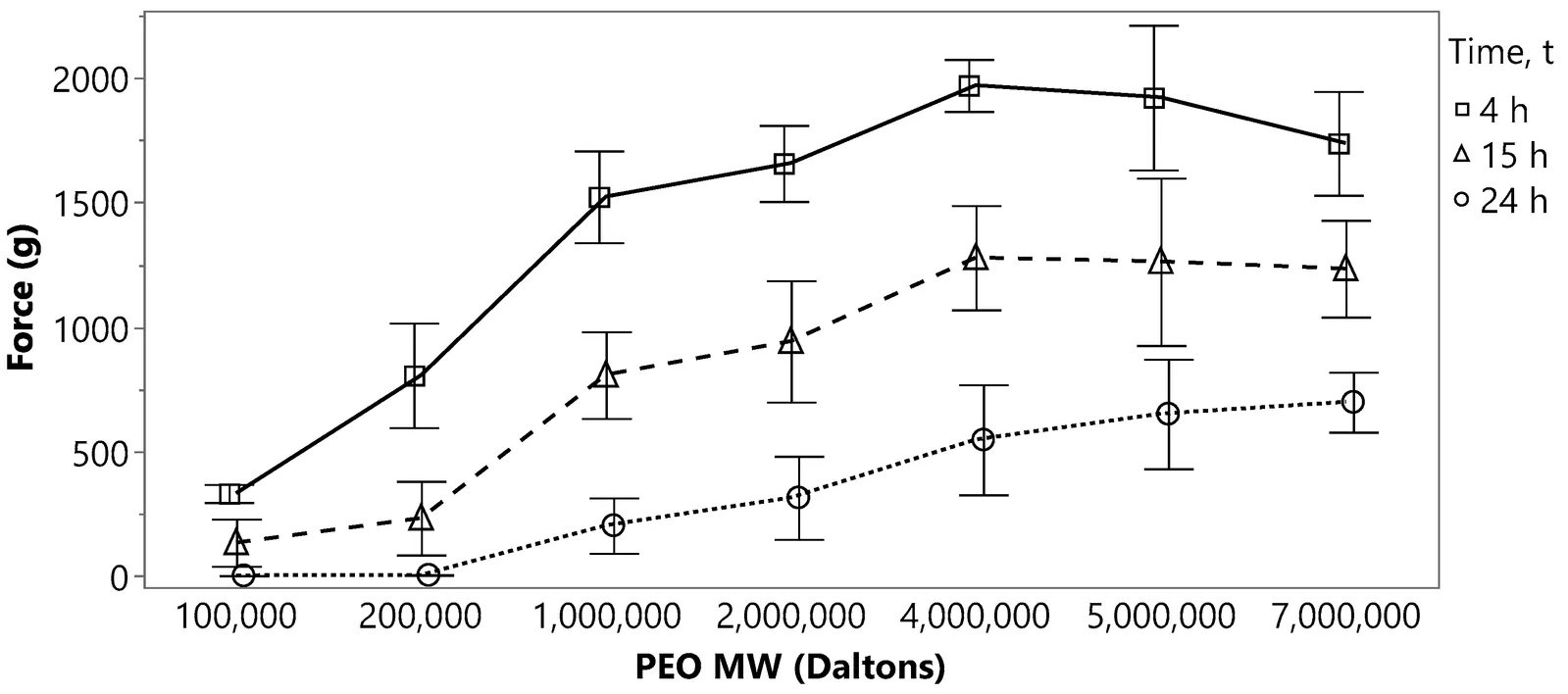
Push-layer swelling forces at 4, 15, and 24 h as functions of PEO MW grade.

The relationship between push-layer swelling force and PEO MW is further demonstrated in Figure 8, where for the 4 and 15 h data (F_s_4h and F_s_15h), push-layer swelling force increased in nearly linear fashion from PEO 100,000 up to PEO 4,000,000, and then essentially plateaued with the three high MW grades. The 24 h swelling-force data (F_s_24h) increased linearly from PEO 200,000 up to PEO 7,000,000, but the differences in F_s_24h generated by the high MW grades were not statistically significant (*p* > 0.05).

**Figure 9:**
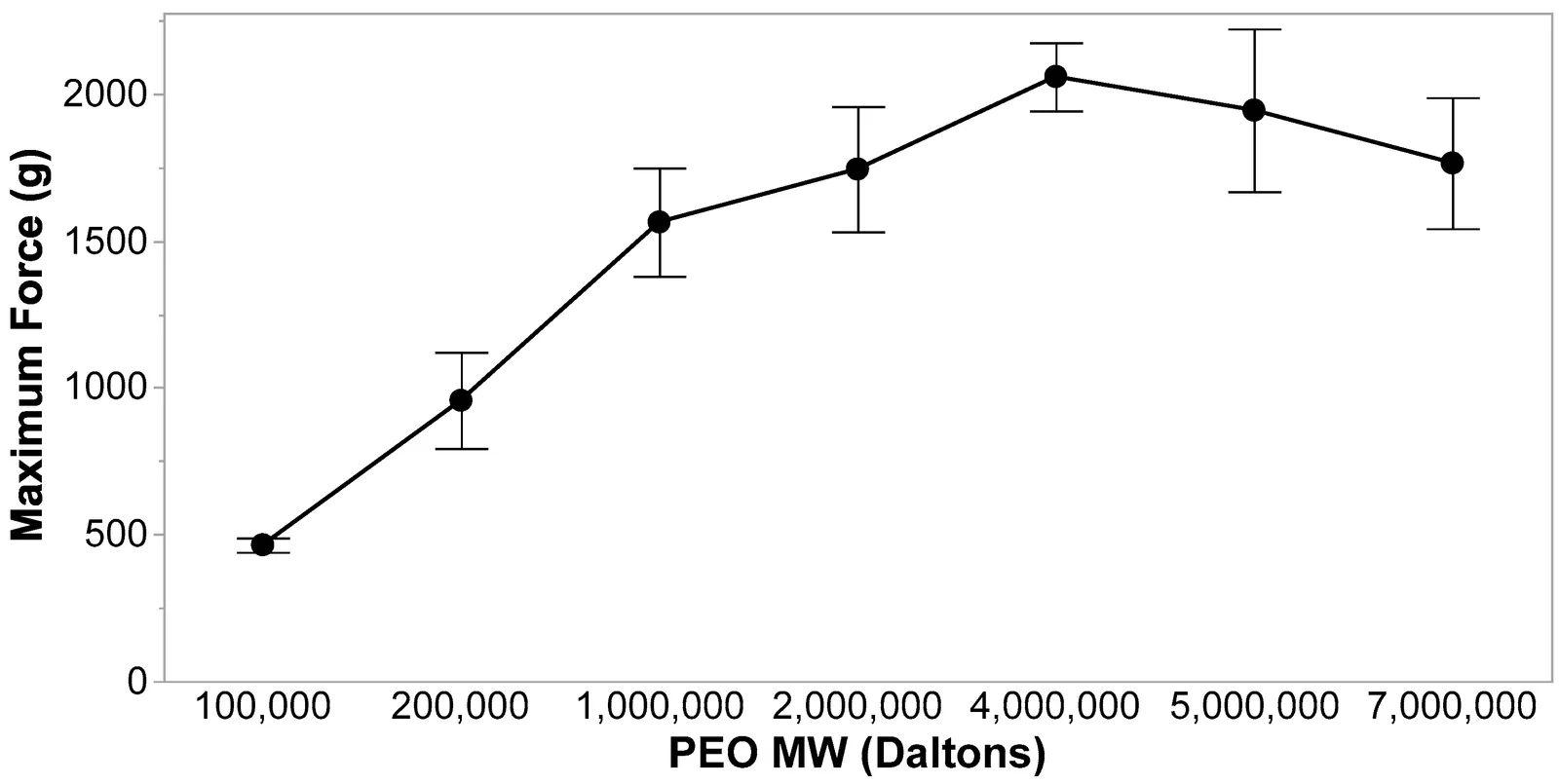
Maximum swelling force as a function of the PEO MW grade in the push layer.

Maximum swelling force (F_s,max_) values are plotted in Figure 9 vs. PEO MW grade. These data also reflect nearly linear increase in F_s,max_ with increasing MW grade from PEO 100,000 up to PEO 4,000,000, followed by a plateau or slight decline in F_s,max_ beyond PEO 4,000,000. There was no statistically significant difference (*p* > 0.05) in F_s,max_ from push layers containing the 2, 4, 5, and 7,000,000 MW grades. Therefore, we hypothesize that, while maximum swelling force likely impacts controlled-release performance, the extent to which swelling force can be sustained over the entire 24 h dosage interval may be more impactful. Hence, other swelling-force profile parameters were subjected to statistical analysis, particularly areas under the swelling-force curve (AUC_Fs_).

Listed in Table 3 are the swelling-force parameters attained from push layers containing the various PEO MW grades. F_s_15h, F_s,max_, AUC_Fs,0–15h_, and AUC_Fs,0–24h_ numerically describe swelling-force parameter increases from push layers containing PEO 100,000 up to PEO 4,000,000, as well as the similarities in parameters from push layers containing PEO 4,000,000 and greater. Only the F_s_24h data indicate an increase in swelling force from PEO 100,000 up to PEO 7,000,000. Again, the differences in F_s_24h data did not reach statistical significance beyond PEO 4,000,000.

**Figure 10:**
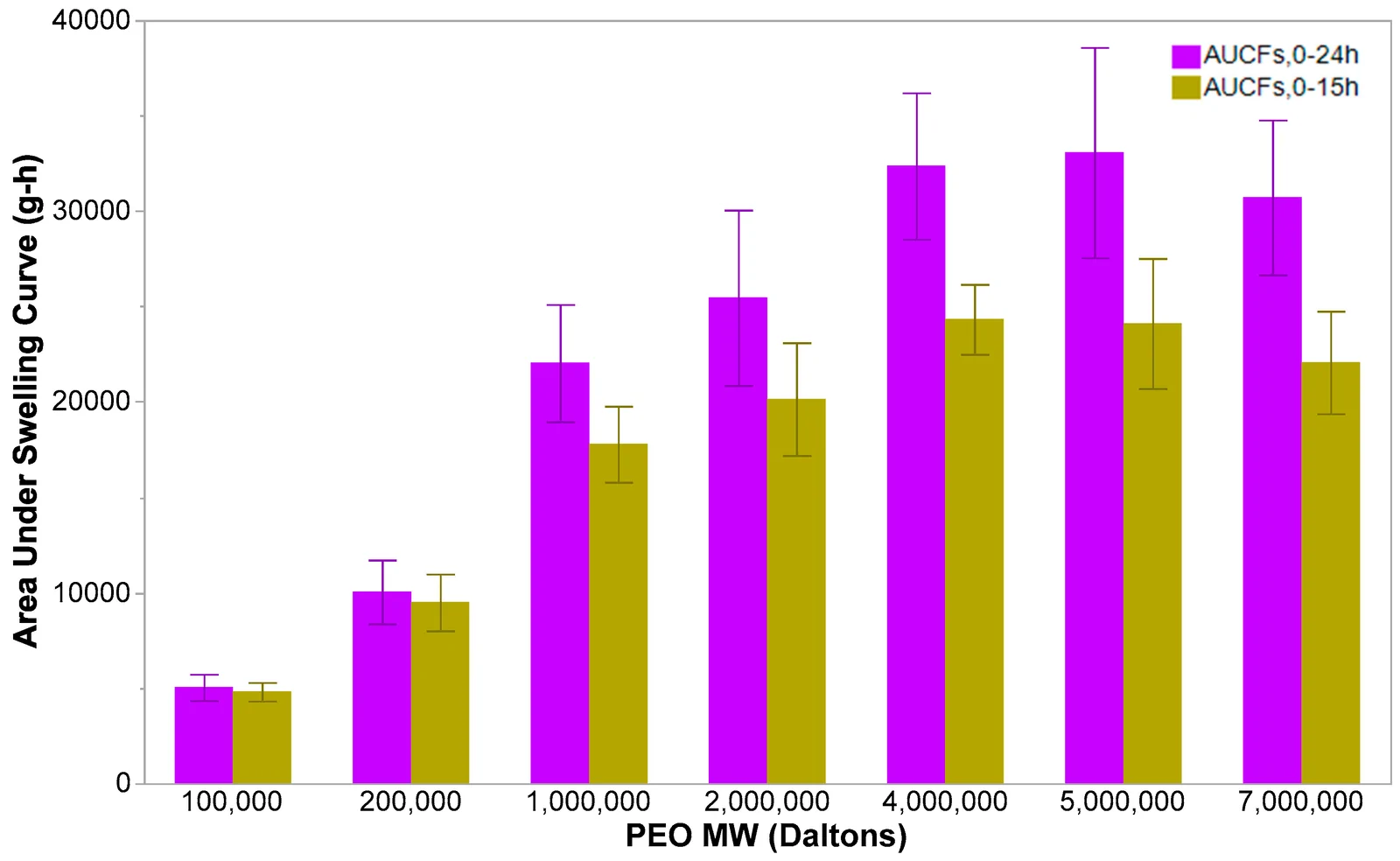
Areas under the swelling force vs. time profiles as functions of the PEO MW grade in the push layer. Area under the swelling-force curve was calculated between 0 and 24 h (purple) and between 0 and 15 h (olive green).

**Figure 11:**
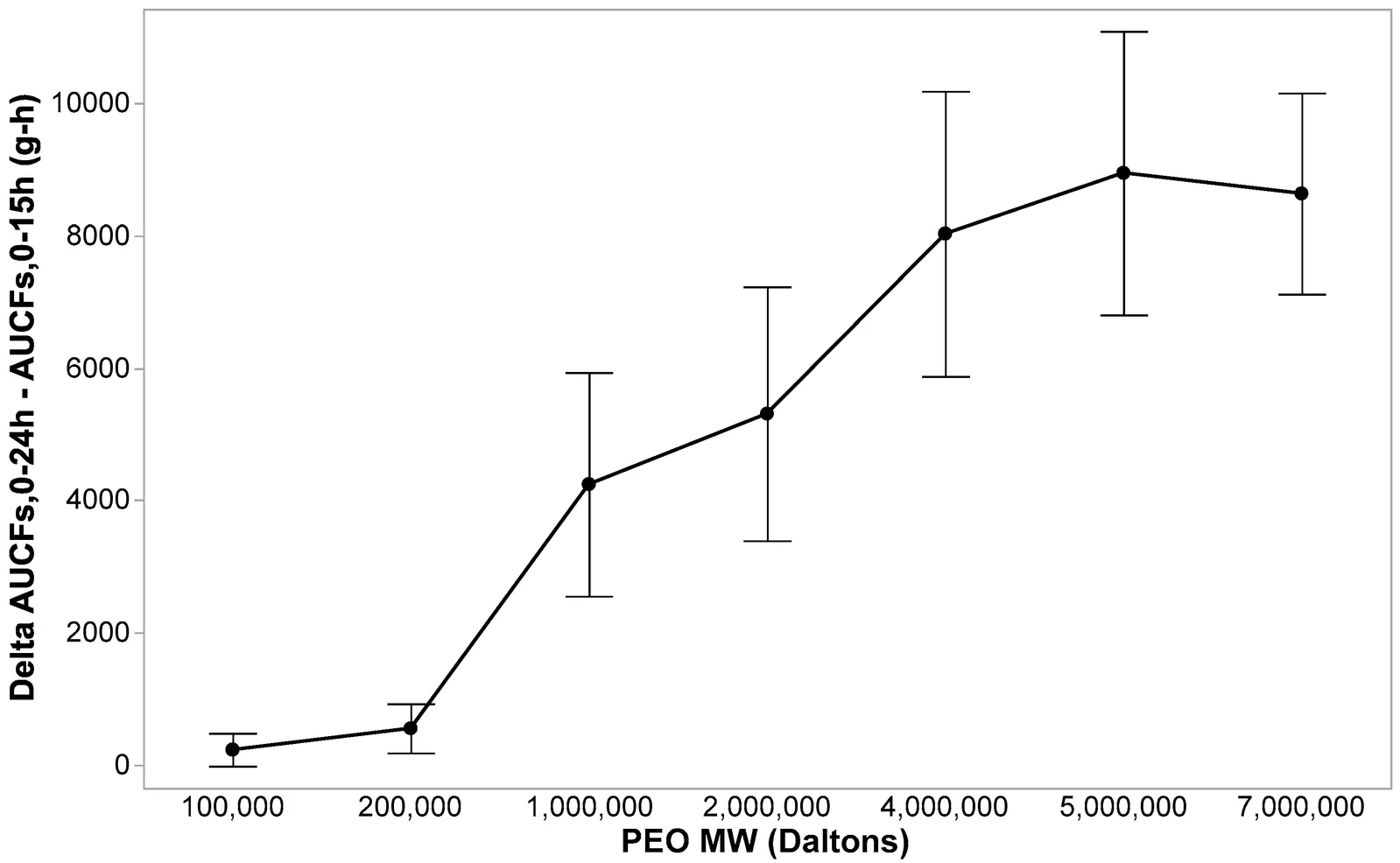
Delta (i.e., difference) between areas under the swelling force vs. time profiles, AUC_Fs,0–24h_—AUC_Fs,0–15h_, as a function of the PEO MW grade in the push layer.

Areas under the swelling-force curves between 0 and 15 h and 0 and 24 h were quantified to obtain single numerical parameters for swelling force attained from the push layers over those respective timeframes. In addition to the AUC_Fs,0–15h_ and AUC_Fs,0–24h_ parameters, the greater the delta between AUC_Fs,0–15h_ and AUC_Fs,0–24h_, the greater the swelling force enabled by the respective PEO MW grade between 15 and 24 h. Recall from Figure 4 that nifedipine release kinetics transitioned from zero- to first-order between 15 and 16 h and that the remaining 9 mg of the 30 mg nifedipine dosage was released from the PPOP tablet over the last 9 h following that transition in release kinetics. From Figure 10, the three highest PEO MW grades enabled the greatest swelling force over 15 and 24 h, which could be mechanistically important for a controlled-release OSDF designed to deliver API over an entire 24 h duration. Referring to Figure 11, the deltas between unrounded AUC_Fs,0–15h_ and AUC_Fs,0–24h_ values from push layers containing the PEO 4, 5, and 7,000,000 MW grades were calculated to be 8035, 8602, and 8643 g-h, respectively. Hence, we hypothesize, based upon the present dataset, that a swelling-force AUC_Fs_ of at least 8000 g-h between 15 and 24 h is necessary to deliver the final 9 mg of the nifedipine dosage over those last 9 h. The corresponding deltas from push layers containing PEO 100,000, 200,000, 1,000,000, and 2,000,000 MW grades were 225, 493, 4243, and 5311 g-h, respectively. Hence, only the three highest PEO MW grades delivered the needed swelling force during the latter portion of the dosage interval.

## 4. Discussion

A PPOP tablet relies on osmotic pressure for drawing water into its core to activate swelling. The osmotic pressure gradient is enabled by including an osmogen in the core [6]. The polymer in the PPOP push layer provides the swelling force needed to drive the drug layer out of the laser-drilled orifice in controlled fashion. The MW of the polymer dictates push-layer swelling. The barrier membrane coating constrains the push layer to swell in the axial dimension, which provides the piston-like driving force that pushes the drug layer out of the laser-drilled orifice. Being able to quantify swelling force in the axial dimension over time provides mechanistic insight into the driving functionality provided by the PPOP push layer.

Developing this methodology resulted in a discerning technique for measuring push-layer swelling force. The methodology not only distinguished swelling force attained across several of the PEO MW grades; it also provided quantitative swelling-force data justifying why the three highest PEO MW grades are recommended for the PPOP push layer. PPOP tablets are often designed to be administered once daily and deliver API in extended and consistent fashion until administration is repeated 24 h later. Hence, the push layer should provide sufficient and sustainable swelling force needed for controlled-release functionality over the entire dosage interval. The deltas between the AUC_Fs_ swelling-force parameters, AUC_Fs,0–24h_—AUC_Fs,0–15h_, quantitatively justify why the three highest PEO MW grades are recommended in the PPOP push layer. Only these high-MW PEO grades deliver sufficient swelling force to drive the remaining nifedipine dosage from the PPOP tablet during those final hours of the dosage interval.

Finally, developing this methodology has advanced the fundamental understanding of PPOP push-layer functionality, but we should also objectively communicate both the shortcomings of the methodology at its current state of development and recommendations for future studies. Each set of testing conditions was repeated seven times (*n* = 7), with each individual test lasting 24 h. The study duration was lengthy due to the use of a single texture analyzer. The data provided by texture analysis were extensive at a capture rate of one measurement per second, which challenged the computational capacity of Microsoft Excel. We evaluated swelling force only in water for initial method development, the justification being that PEO is a nonionic polymer, which does not exhibit pH-dependent solubility. Regardless, future studies should include physiologically relevant buffers. We did not control water temperature, allowing it to vary with the ambient conditions of the laboratory, because PEO does not exhibit thermogelation properties like some cellulose ethers do, for example. In the future, we should fabricate a temperature-controlled environment around the measurement setup that will allow equilibration and testing at 37 °C over 24 h, without interfering with the measurement equipment functionality. Furthermore, the gap between the cylindrical wall and the texture-analyzer probe could conceivably allow a minor extent of push-layer erosion. The higher the molecular weight of PEO, the greater the chain entanglement, forming a thicker, more mechanically robust gel layer. In contrast, the lower PEO MW grades produce fewer chain entanglements upon swelling, resulting in weaker gel layers that are more prone to chain disentanglement and erosion. Future studies should evaluate how much PEO is lost due to erosion, as well as the impact erosion could have on the swelling-force measurement. Finally, we fabricated the Setup 3 configuration with plastic rather than with a semipermeable material, such as the barrier membrane made from cellulose acetate. Fabricating the holder using plastic allowed adjustment of the setup without compromising reproducible and rigid confinement for dimensional swelling control. Future studies should utilize Setup 3 fabricated with the same material that forms the barrier membrane around the bilayer tablet core.

With these shortcomings and proposed future studies communicated, this is a first step in being able to quantify the crucial pushing force mechanism that drives PPOP tablet performance over extended dosage intervals. Conducting future studies to advance the methodology is clearly worthwhile and should be done prior to validating this new measurement methodology. That stated, our immediate next step involves shifting focus to more fundamentally and quantitatively characterizing the PPOP pull layer.

## 5. Conclusions

We have developed first-generation measurement methodology for quantitatively understanding push-layer swelling force and fundamentally explaining the driving mechanism of PPOP tablet functionality. The methodology quantifies the force needed to drive the API dosage from the PPOP tablet over an extended duration, such as 24 h. More specifically, one can now quantitatively explain why PEO is used in the PPOP push layer and what PEO MW grades will deliver the needed swelling force for the necessary duration. Even at its early stage of development, the methodology demonstrated, in discriminatory fashion, that push-layer swelling force increased from PEO 100,000 up to PEO 4,000,000; and then it plateaued for the three highest MW grades, PEO 4, 5, and 7,000,000.

Based on our learnings using this new quantitative methodology, we hypothesize the mechanistic relationship between controlled release from the PPOP tablet over the entire dosage interval and push-layer swelling functionality over that same timeframe. The method does still require further development and optimization before it could be considered for validation, but it already shows promise at quantifying mechanistic functionality of the PPOP push layer.

## Figures and Tables

**Table 1:** Marketed PPOP tablet formulations.

Product	Active Pharmaceutical Ingredient	Company	Treatment Type
Procardia XL	Nifedipine	Pfizer/ALZA	Antihypertensive
Adalat CC	Nifedipine	Bayer	Antihypertensive
Glucotrol XL	Glipizide	Pfizer/ALZA	Antidiabetic
Ditropan XL	Oxybutynin	ALZA	Overactive bladder
Concerta	Methylphenidate	Janssen/ALZA	Attention-Deficit Hyperactive Disorder
Invega	Paliperidone	Janssen	Antipsychotic
Cardura XL	Doxazosin	Pfizer	Benign Prostatic Hypertrophy/Antihypertensive
Covera-HS	Verapamil	Searle/ALZA	Antihypertensive
Jurnista/Exalgo	Hydromorphone	ALZA/Janssen	Pain management

**Table 2:** Materials used to formulate push-layer compacts.

Material	Brand Name (If Applicable)	Lot Number	Supplier
PEO 100,000 ^i^	POLYOX™ WSR N10	2744225711	Roquette (Institute, WV USA)
PEO 200,000 ^i^	POLYOX™ WSR N80	2744703171	Roquette (Institute, WV USA)
PEO 1,000,000 ^i^	POLYOX™ WSR N-12K	2744209048	Roquette (Institute, WV USA)
PEO 2,000,000 ^i^	POLYOX™ WSR N-60K	D682L8VPM5	Roquette (Institute, WV USA)
PEO 4,000,000 ^i^	POLYOX™ WSR 301	2744613478	Roquette (Institute, WV USA)
PEO 5,000,000 ^i^	POLYOX™ WSR Coagulant	2744659049	Roquette (Institute, WV USA)
PEO 7,000,000 ^i^	POLYOX™ WSR 303	2744666856	Roquette (Institute, WV USA)
Micronized NaCl ^ii^	Micro 95 1.5% TCP ^iii^	0041399972	Cargill (Akron, OH USA)
Red Fe_2_O_3_^iv^		P28K015	Thermo Scientific (Allentown, PA USA)

^i^ Polyethylene oxide approximate MW grade (Daltons). ^ii^ Sodium chloride. ^iii^ 95% of the NaCl is less than 44 microns. ^iv^ Iron oxide.

**Table 3:** Quantitative parameters from swelling force vs. time profiles.

PEO MW Grade ^i^ in Push Layer	F_s_15h ^ii^	F_s_24h ^iii^	F_s,max_^iv^	AUC_Fs,0–15h_^v^	AUC_Fs,0–24h_^vi^
PEO 100,000	134 ± 95	3 ± 1	464 ± 24	4811 ± 489	5036 ± 693
PEO 200,000	233 ± 148	5 ± 2	956 ± 164	9566 ± 1620	10,059 ± 1705
PEO 1,000,000	808 ± 174	204 ± 111	1563 ± 184	17,794 ± 1993	22,037 ± 3074
PEO 2,000,000	943 ± 243	315 ± 167	1743 ± 213	20,153 ± 2957	25,464 ± 4589
PEO 4,000,000	1279 ± 209	548 ± 221	2058 ± 116	24,332 ± 1827	32,368 ± 3838
PEO 5,000,000	1263 ± 336	652 ± 220	1943 ± 277	23,569 ± 4262	32,171 ± 7003
PEO 7,000,000	1235 ± 194	699 ± 121	1764 ± 223	22,075 ± 2683	30,719 ± 4063

^i^ Polyethylene oxide MW grade (Daltons). ^ii^ Swelling force at 15 h (g). ^iii^ Swelling force at 24 h (g). ^iv^ Maximum swelling force (g). ^v^ Area under swelling-force curve 0–15 h (g–h). ^vi^ Area under swelling-force curve 0–24 h (g–h).

## Data Availability

The original contributions and data presented in this study are included in the figures and tables of this article. Further inquiries can be directed to the corresponding author.

## Abbreviations

The following abbreviations are used in this manuscript:

API	Active pharmaceutical ingredient
AUC_Fs,0–15h_	Area under swelling-force curve from 0 to 15 h
AUC_Fs,0–24h_	Area under swelling-force curve from 0 to 24 h
F_s_15 h	Swelling force at 15 h
F_s_24 h	Swelling force at 24 h
F_s,max_	Maximum swelling force
Fe_2_O_3_	Iron oxide
FFBE	Flat-face beveled-edge
g	Gram
h	Hours
HPLC	High-performance liquid chromatography
HSD	Honestly significant difference
kg	Kilogram
mL	Milliliter
mm	Millimeter
MW	Molecular weight
n	Number of repetitions
NaCl	Sodium Chloride
OSDF	Oral solid dosage form
PEO	Polyethylene oxide
PPOP	Push–Pull osmotic pump
UV	Ultraviolet
